# Transcriptome analysis and identification of genes related to terpenoid biosynthesis in *Cinnamomum camphora*

**DOI:** 10.1186/s12864-018-4941-1

**Published:** 2018-07-24

**Authors:** Caihui Chen, Yongjie Zheng, Yongda Zhong, Yangfang Wu, Zhiting Li, Li-An Xu, Meng Xu

**Affiliations:** 1 Co-Innovation Center for Sustainable Forestry in Southern China, Nanjing Forestry University, Nanjing, 210037 China; 2 Camphor Engineering Technology Research Center for State Forestry Administration, Jiangxi Academy of Forestry, Nanchang, 330032 China; 3 Institute of Biological Resources, Jiangxi Academy of Science, Nanchang, Jiangxi China

**Keywords:** Terpenoids, Terpenoid biosynthesis, *Cinnamomum camphora*, Transcriptome, Different chemotypes

## Abstract

**Background:**

*Cinnamomum camphora* has been cultivated as an economically important tree for its medicinal and aromatic properties. Selective breeding has produced *Cinnamomum* plants for special uses, including spice strains with characteristic flavors and aromas and high-potency medicinal cultivars. The molecular biology underlying terpenoid biosynthesis is still unexplored.

**Results:**

Gas chromatography-mass spectrometry was used to analyze the differences in contents and compositions of essential oil terpenoids in linalool- and borneol-type chemotypes of *C. camphora*. The data revealed that the essential oils consist primarily of monoterpenes with only very minor quantities of sesquiterpenes and diterpenes and that the essential oil differs in different chemotypes of *C. camphora*, with higher yields of (−)-borneol from the borneol-type than from the linalool-type. To study the terpenoid biosynthesis of signature compounds of the major monoterpenes, we performed RNA sequencing to profile the leaf transcriptomes of the two chemotypes of *C. camphora*. A total of 23.76 Gb clean data was generated from two chemotypes and assembled into 156,184 unigenes. The total length, average length, N50 and GC content of unigenes were 155,645,929 bp, 997 bp, 1430 bp, and 46.5%, respectively. Among them, 76,421 unigenes were annotated by publicly available databases, of which 67 candidate unigenes were identified to be involved in terpenoid biosynthesis in *C. camphora*. A total of 2863 unigenes were identified to be differentially expression between borneol-type and linalool-type, including 1714 up-regulated and 1149 down-regulated unigenes. Most genes encoding proteins involved in terpenoid precursor MVA and MEP pathways were expressed in similar levels in both chemotypes of *C. camphora*. In addition, 10 and 17 DEGs were significantly enriched in the terpene synthase activity and oxidoreductase activity terms of their directed acyclic graphs (DAG), respectively. Three monoterpene synthase genes, *TPS14-like1, TPS14-like2* and *TPS14-like3* were up-regulated in the borneol-type compared to the linalool-type, and their expression levels were further verified using quantitative real-time PCR.

**Conclusions:**

This study provides a global overview of gene expression patterns related to terpenoid biosynthesis in *C. camphora*, and could contribute to a better understanding of the differential accumulation of terpenoids in different *C. camphora* chemotypes.

**Electronic supplementary material:**

The online version of this article (10.1186/s12864-018-4941-1) contains supplementary material, which is available to authorized users.

## Background

*Cinnamomum camphora*, a member of the Lauraceae family, is an evergreen broad-leaf tree indigenous to southern China and Japan [1]. The essential oil or crystal distilled from *C. camphora* has considerable economic importance as a source of food preservative and additive, and as raw materials for the cosmetic and pharmaceutical industries. The chemical compositions of different *C. camphora* tissues have been previously investigated, and mono- and sesquiterpenes were found abundant in the leaves and twigs [2, 3]. There is chemical polymorphism in *C. camphora*. According to the signature constituent in leaf, *C. camphora* was classified into at least five different chemical variants (chemotypes), including linalool- (58–92%), borneol- (67–82%), camphor- (54–97%), cineole- (32–52%), and nerolidol-types (16–57%) [4, 5]. In recent years, *C. camphora* has become of increasing importance as a source of essential oils, especially for the production of natural borneol and linalool [6]. Borneol, a bicyclic monoterpene alcohol, has been widely used in food and drug industries typically in folk medicine in China and India. Interestingly, natural borneol has been used to increase the permeabilities of the intestinal mucosa and blood-brain barrier to improve the oral bioavailability of some poorly permeable drugs [7].

Terpenoids (or terpenes) constitute the largest class of structurally diverse metabolites, with more than 55,000 members identified in living organisms [8, 9]. Based on the number of 5-carbon units, terpenoids are classified into monoterpene (C10), sesquiterpenes (C15), diterpenes (C20), triterpenes (C30), tetraterpenes (C40) and polyterpenes which have more than eight 5C units [10]. Borneol and linalool, like all other monoterpenes, are formed from the isomeric 5-carbon building blocks isopentenyl diphosphate (IPP) and dimethylallyl diphosphate (DMAPP). Two independent pathways participate in the biosynthesis of IPP and DMAPP in plants. In the 2-C-methyl-D-erythritol 4-phosphate (MEP) pathway, the biosynthesis of IPP/DMAPP in plastids begins with pyruvate and glyceraldehyde-3-phosphate [11, 12], whereas in the cytosol and peroxisomes, IPP/DMAPP formation occurs from the condensation of acetyl-CoA in the mevalonate acid (MVA) pathway [13, 14]. The equilibrium of IPP and DMAPP is controlled by IPP Delta-isomerase (IDI), which reversibly converts IPP to DMAPP [15]. The condensation of the C5 precursors leads to the formation of monoterpenes, sesquiterpenes, and diterpenes by the activation of terpene synthases (TPS) [16, 17]. Terpenoid synthesis is often correlated with the induction of TPS gene expression [18–20] and several monoterpene and sesquiterpene synthases have been isolated and characterized from various plant species [21, 22]. Besides the regulation at the level of terpene synthase activity, the induction of precursor biosynthetic genes has also been described [23, 24].

Recently, the development of RNA-sequencing (RNA-Seq) provided an opportunity for detailed transcriptomic studies, even in species without a reference genome [25]. De novo transcriptome assembly has been widely applied to investigate and identify the critical genes involved in the biosynthesis of secondary metabolites in medicinal and aromatic plants, such as *Artemisia annua* [26], *Mentha spicate* [27], *Cinnamomum camphora* [28]*, Salvia miltiorrhiza* [29], *Lindera glauca* [30] and *Huperzia serrata* [31].

In light of the chemical polymorphism evident in *C. camphora*, it is of interest to search for potential differences in the terpenoid biosynthesis pathway in different chemotypes. We have used metabolic analysis and transcriptome sequencing in order to discover potential monoterpene synthases present in *C. camphora* chemotypes. In the current study, the leaf contents and compositions of terpenoids in borneol- and linalool-types was analyzed with gas chromatography-mass spectrometry (GC-MS) and their transcriptomes compared. After functional annotation and classification, the genes involved in the MEP/MVA pathways and terpene synthesis in both chemotypes were identified. The results contribute to our understanding of the mechanism underlying differences in terpenoid biosynthesis between *C. camphora* chemotypes.

## Methods

### Plant materials

**Fig. 1:**
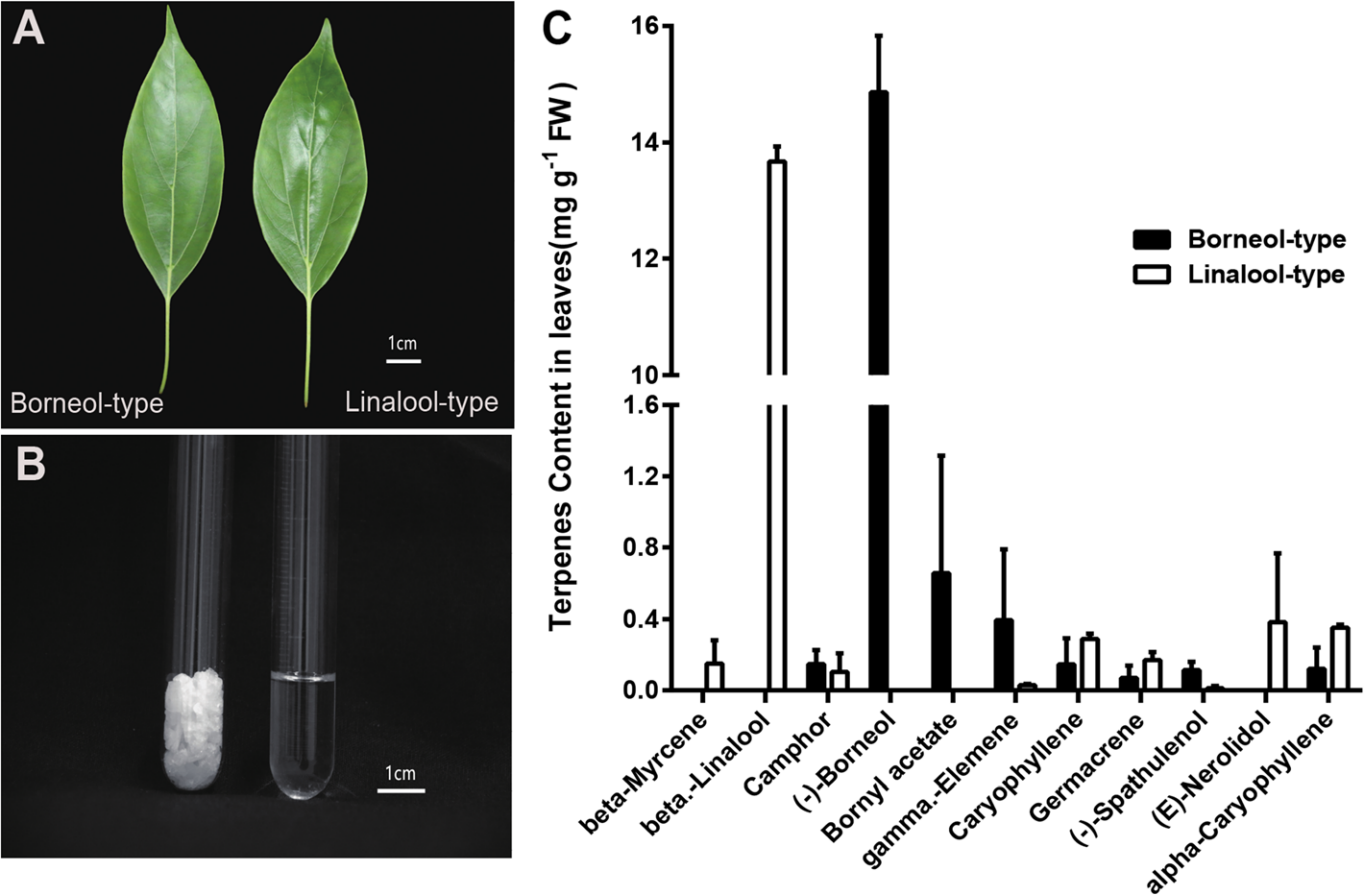
Composition of the leaf extracts of borneol-type and linalool-type of *C. camphora*. **a** *C. camphora* leaves used in transcriptome sequencing. **b** Leaf extracts obtained by water-distillation from *C. camphora* (left borneol-type, right linalool-type). The scale bar =1 cm in A and B. **c** Terpene composition obtained by GC-MS from borneol and linalool chemotype leaves of *C. camphora*. FW: fresh weight. The terpenes content > 0.1 mg. g^− 1^ FW are shown for simplicity

Leaves of linalool- (No. JF1, JF2) and borneol-type (No. JL1, JL4) *C. camphora* were collected for chemical analysis and RNA sequencing from trees grown at the experimental tree farm of the Jiangxi Academy of Forestry in Nanchang, China (Fig. 1a). Two 8-year-old trees of each chemotype were used as biological replicates. The two replications for the linalool chemotype were termed F_L1 and F_L2, and the borneol chemotype were termed L_L1 and L_L2. Three chemotypes of *C. camphora* (camphor-, cineole- and nerolidol-types) were obtained from the campus of Nanjing Forestry University for further analysis, the compositions of their leaf extracts are listed in Additional file 1. All the samples for RNA extraction were frozen in liquid nitrogen immediately and stored at − 80 °C. At the same time, fresh leaves of different chemotypes were obtained for essential oil isolation.

### Measurement of leaf terpenoids

The fresh leaves of each sample were hydrodistilled with a modified Clevenger-type apparatus for 2 h until the extraction was completed. The terpenoid composition analysis of each sample were performed on a SHIMADZU QP2020 gas chromatograph-mass spectrometer (SHIMADZU Corporation, Japan), fitted with a DB-5-MS silica capillary column (30 m × 0.32 mm, 0.25 μm film thickness). Helium was used as the carrier gas with a constant flowrate of 0.6 mL/min. The GC temperature program were as follows: 50 °C for 3 min, a gradient of 50–180 °C over 16.25 min followed by holding at 180 °C for 1 min, then a gradient of 180–280 °C over 10 min followed by holding at 280 °C 5 min. Alkanes were used as reference points in the calculation of relative retention indices. The GC inlet was operated at 280 °C in splitless mode with 0.6 μL injection volume. The quadrupole MS operating parameters were set as follows: electron ionization (EI) mode; EI source, 70 eV; transfer line temperature, 250 °C; ion source temperature, 200 °C; emission current, 150 μA; examination voltage, 500 V; 0.2 s for the full scan mode; scan mass range, 29–450 m/z. The peaks were identified by comparing their retention time with that of the known standards, which were determined under the same conditions. A library search was carried out using the Wiley GC-MS Library and the TBAM Library of Essential Oil Constituents.

### Total RNA extraction and transcriptome sequencing

Total RNAs from all samples were extracted using the RNeasy Plant Mini Kit (Qiagen, Hilde, Germany), and immediately frozen at − 80 °C until use. RNA degradation and contamination were monitored on 1% agarose gels. RNA integrity was assessed using the RNA Nano 6000 Assay Kit of the Agilent Bioanalyzer 2100 system (Agilent Technologies, CA, USA). A total amount of 1.5 μg of RNA extracted from each leaf sample was used for transcriptome analysis using Illumina’s kit (Illumina, San Diego, CA, USA) following the manufacturer’s protocol. Four sequencing libraries were generated using the NEBNext® Ultra™ RNA Library Prep Kit for Illumina® (NEB, Boston, Massachusetts, USA) following the manufacturer’s recommendations and index codes were added to attribute sequences to each sample. The cDNA library preparations were sequenced on an Illumina HiSeq 2000 platform (Illumina, San Diego, CA, USA) and paired-end reads were generated.

### De novo assembly and annotation

For the assembly library, raw data (raw reads) in FASTQ format were first processed through CASAVA base-calling. Trimmomatic (version 0.36) [32] were used to trim and crop FASTQ data as well as to remove adapters. The parameters of removing reads containing N or low quality (reads containing more than 50% bases with Q-value ≤20) were default with paired end mode. All the libraries were mixed together to generate an assembly using Trinity (version r20140413pl) [33], and default parameters were used except for the minimum kmer coverage setting to 2. The assembly results were further clustered and filtered to get the non-redundant unigenes by Corset (version 1.07) [34]. Gene function was annotated using BLAST program with an E-value cut-off of 1e^− 5^ against the following databases: NCBI non-redundant protein sequences (Nr, https://blast.ncbi.nlm.nih.gov/), NCBI non-redundant nucleotide sequences (Nt, https://blast.ncbi.nlm.nih.gov/), Protein family (Pfam, http://pfam.sanger.ac.uk/), and EuKaryotic Orthologous Groups (KOG) database (http://www.ncbi.nlm.nih.gov/COG/). Functional annotation by Gene ontology (GO) terms was analyzed by Blast2GO version 2.5 [35] (http://www.geneontology.org). The annotation of gene association with the Kyoto encyclopedia of genes and genomes (KEGG) pathways was performed by KAAS (r140224).

### Analysis of the differential expression of unigenes

The number of fragments per kilobase of exon per million fragments mapped (FPKM) was used to estimate the relative expression levels [36] and the FPKM of unigenes were estimated using the software RSEM v1.2.15 [37], with the Trimmed Mean of M-values method. Differential expression analysis of two chemotypes of *C. camphora* samples was performed using read counts with the DESeq R package (v1.10.1) [38]. The resulting *P*-values were adjusted to Q-values to compensate for multiple hypothesis testing [39]. Genes with an adjusted Q-value < 0.05 found by DESeq were assigned as differentially expressed. GO enrichment analysis of differentially expressed genes (DEGs) were performed using the GOseq R package (version 1.10.0) based on the Wallenius non-central hypergeometric distribution, which can adjust for gene length bias in DEGs [40]. KEGG enrichment analysis was performed with KOBAS version 2.0.12 software [41].

### Validation of DEGs by qRT-PCR analysis

Quantitative reverse transcription polymerase chain reaction (qRT-PCR) was employed using the SYBR Green PCR Master Mix (Takara, Dalian, China) and the ABI ViiA 7 Real-time PCR platform. The primers were aligned to the target gene sequence using Oligo 7.0 (Molecular Biology Insights, Cascade, CO, USA). The qRT-PCR analyses were performed for the following genes: *GPS_like* (Cluster-13,185.62746), *HMGR_like* (Cluster-13,185.61515), *HDS_like1* and *HDS_like2* (Clusters-13,185.59980 and 13,185.80000, respectively), *HDR* (Cluster-13,185.61134), *DXR_like* (Cluster-13,185.63961), *Mg17_like* (Cluster-13,185.50937), *TPS14_like1*, *TPS14_like2* and *TPS14_like3* (Clusters-13,185.98748, 13,185.75128 and 13,185.81558, respectively), *SDR_like1* and *SDR_like2* (short-chain dehydrogenases, Clusters 13,185.106850 and 13,185.105357) and one putative linalool synthase gene were submitted to GenBank (accession no. XJ028228, *C. camphora*). Details of the primers used for the qRT-qPCR assay are listed in Additional file 2, and sequences of these unigenes are listed in Additional file 3. The experiments were performed with three technical replicates that contained 30 ng cDNA, 10 μL of SYBR Green PCR Master Mix, and 600 nM primers and nuclease free water with a final volume of 20 μL per reaction. The Ct values for all genes were normalized to the Ct value of actin (*ACT*, KM086738.1).

## Results

### The composition of leaf extracts from different chemotypes of *C. camphora*

Specimens of *C. camphora* leaves from two chemotypes (F_L1 and F_L2 were linalool-types, L_L1 and L_L2 were borneol-types) were obtained from the garden of Jiangxi Academy of Forestry and the leaf extracts were isolated by water distillation. There were no morphological differences in between the leaves from the two chemotypes of *C. camphora* (Fig. 1a). Interestingly, the leaf essential oil extracts of the linalool-type remained as oil liquids, whereas that of the borneol-type formed crystals (Fig. 1b). The compositions of leaf essential oil from the fresh leaves were analyzed with GC-MS. A total of 24 terpenoid compounds in leaf distillates were detected, and monoterpenes were the main constituents (Table 1). The major components with yields > 0.1 mg.g^− 1^ fresh weight are shown in Fig. 1c. In the extracts of both chemotypes, the signature compound was present in far higher content than all other compounds. In the linalool-type, the most abundant compound, beta-linalool, yielded 13.67 mg.g^− 1^ FW, followed by (E)-nerolidol in yields of 0.38 mg g^− 1^ FW. The principal constituent of the borneol-type was (−)-borneol (14.86 mg g^− 1^ FW), followed by borneol acetate (0.65 mg g^− 1^ FW). To investigate the biosynthesis of major compounds, we proceeded with the analysis of the transcriptomes of the two *C. camphora* chemotypes.

**Table 1:** Composition of the leaf extracts of *C. camphora*

RI^a^	Component	L_L1 (%)	L_L2 (%)	F_L1 (%)	F_L2 (%)
902	alpha.-Thujene	–	–	–	0.13
964	beta.-Phellandrene	–	–	–	0.11
958	beta-Myrcene	–	–	0.12	1.8
976	beta-Ocimene	–	–	1.17	1.57
1059	Eucalyptol	tr	–	–	0.13
1064	7-Octen-2-ol	–	tr	–	–
1082	beta.-Linalool	–	–	83.44	85.97
1121	Camphor	1.4	0.39	–	1.33
1138	(−)-Borneol	97.73	79.81	–	–
1137	Terpinen-4-ol	–	0.33	tr	0.14
1143	alpha-Terpineol	–	0.88	0.14	0.14
1164	trans-Linalool oxide	–	–	0.18	0.15
1277	Bornyl acetate	–	7.56	–	–
1398	1-methylethenyl	–	0.1	–	tr
1431	gamma.-Elemene	–	4.54	0.13	0.23
1469	Naphthalene	–	–	0.87	0.85
1494	Caryophyllene	–	1.68	1.55	2.04
1507	Caryophyllene oxide	–	0.22	0.15	0.11
1515	Germacrene	–	0.8	0.74	1.38
1522	Cyclohexanemethanol	0.1	–	–	–
1536	(−)-Spathulenol	0.42	0.92	0.15	
1564	(E)-Nerolidol	–	tr	4.71	0.13
1579	alpha-Caryophyllene	–	1.38	2.21	2.14
1593	beta-Eudesmol	0.13	tr	–	–
	total %	99.78	98.61	95.56	98.35
	Oil yield %	1.62	1.74	1.67	1.56

^a^RI, Retention indices calculated against n-alkanes(C-C). tr, trace (< 0.1%). F_L1 and F_L2 were linalool-types, L_L1 and L_L2 were borneol-types

### RNA sequencing and transcriptomic assembly

To identify genes involved in terpenoid biosynthesis in *C. camphora*, four RNA libraries (F_L1 and F_L2 were linalool-types, L_L1 and L_L2 were borneol-types) were prepared and analyzed on Illumina Hiseq2000 platform with a pair-end length of 150 bp. A total of 249.6 million reads were generated from four libraries. After filtering out low quality sequences, approximately 237.6 million clean reads with 46.5% GC content were obtained. The throughput and quality of the RNA-Seq data are included in Table 2. All the clean reads obtained from two different chemotypes were assembled by the Trinity method, resulting in 156,184 unigenes with an N50 length of 1430 bp. The total number of assembled unigenes might be overestimated due to the absence of a reference genome. Additionally, a total of 179,016,590 reads (75.34% of all clean reads) were perfectly mapped (mismatch = 0) to the reference transcriptome by RSEM, which showed that the quality of these mapped genes was sufficient to conduct the subsequent analysis (Table 3).

**Table 2:** Summary of RNA-seq data from four RNA libraries of linalool and borneol chemotypes of *C. camphora*

Libraries	Raw Reads	Clean Reads	Clean Bases	Q30^a^ (%)	GC (%)	Mapped reads
F_L1	66,745,696	63,619,460	9.54G	89.37	45.71	47,532,228(74.71%)
F_L2	61,415,336	58,612,946	8.79G	89.15	45.56	43,734,592(74.62%)
L_L1	61,954,640	59,075,134	8.86G	89.28	45.48	45,017,258(76.20%)
L_L2	59,517,032	56,317,054	8.45G	88.78	45.65	42,732,512(75.88%)
Summary	249,632,704	237,624,594	35.64G			179,016,590(75.34%)

^a^Q30: The percentage of bases with a Phred value > 30

**Table 3:** Length distribution of transcripts and unigenes

Length distribution	Transcripts		Unigenes	
	Number	Percentage (%)	Number	Percentage (%)
201–500	170,861	61.86	53,633	34.34
501–-1000	54,976	19.90	52,225	33.44
1001–2000	32,396	11.74	32,370	20.72
> 2000	17,956	6.50	17,956	11.50
Total	276,189	100.00	156,184	100
Total length (bp)	190,057,842		155,645,929	
Mean length (bp)	688		997	
N50 (bp)	1085		1430	

### Gene annotation and functional classification

**Fig. 2:**
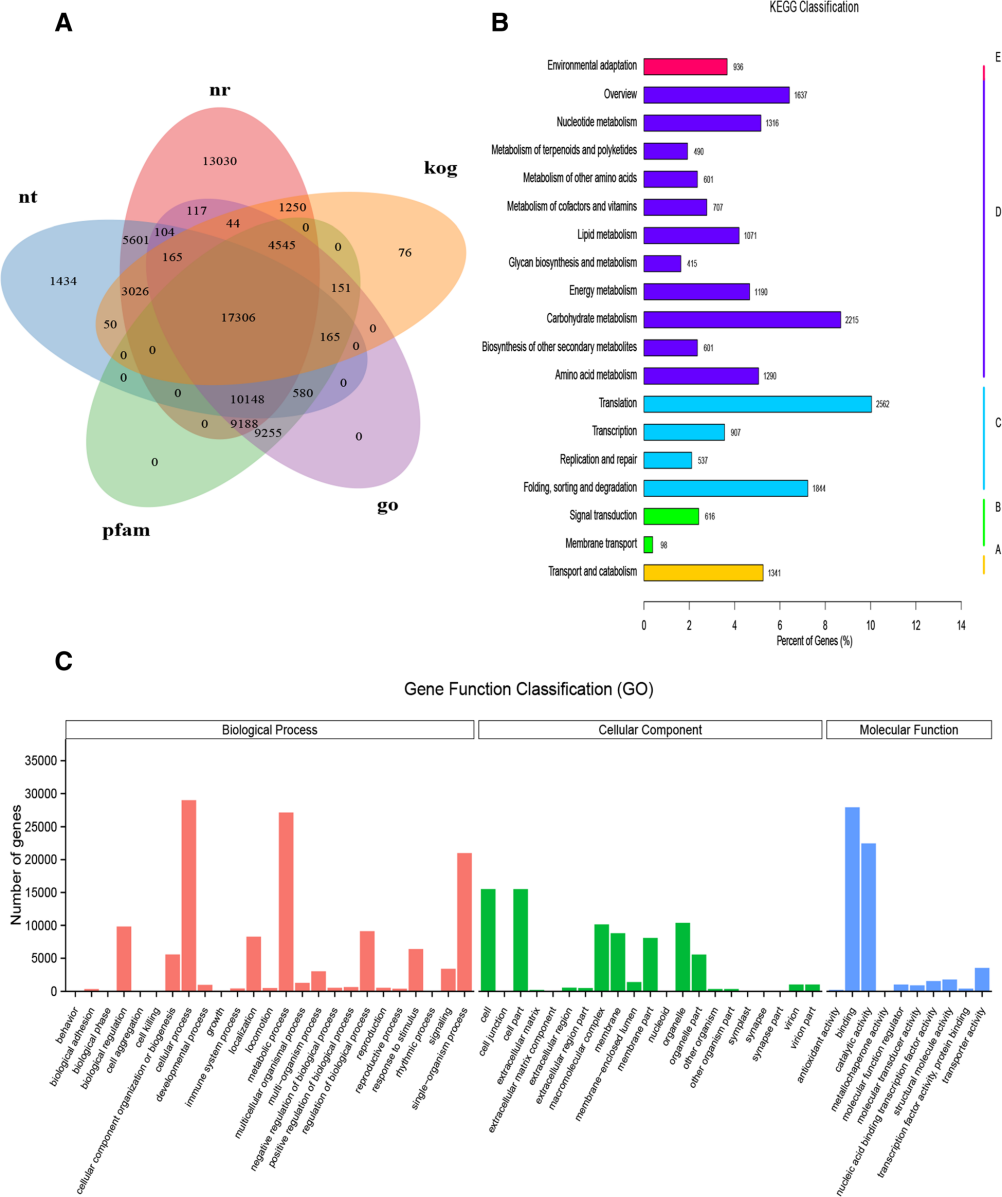
Annotation information of assembled unigenes in *C. camphora*. **a** Venn diagram of the distribution of annotation information from different public databases. **b**. KEGG annotation of putative proteins. The y-axis indicates the name of the KEGG metabolic pathway. The x-axis indicates the percentage of the number of unigenes annotated to the pathway out of the total number of unigenes annotated. The unigenes were divided into five branches according to the KEGG metabolic pathway: Cellular Processes (A), Environmental Information Processing (B), Genetic Information Processing (C), Metabolism (D) and Organismal Systems (E). **c**. GO classification of unigenes in *C. camphora*. Gene Ontology terms are classified into three main categories: biological process (BP), cellular component (CC) and molecular function (MF)

The BLAST alignment was utilized to annotate the 156,184 unigenes of *C. camphora* with an E-value threshold of 1e^− 5^ in the public databases: Nr, Nt, Pfam, GO and KOG. The database annotation results are summarized in Fig. 2a. In summary, 76,070 (48.70%) unigenes were successfully annotated in at least one database, and 17,306 (11.08%) unigenes shared annotation in all databases. There were 64,524 unigenes with significant matches in the Nr database, accounting for the highest proportion (41.31%), while the lowest proportion (26,778; 17.14%) was obtained from the KOG database.

To identify the active biological pathways in *C. camphora*, the assembled unigenes were mapped to the KEGG annotation system (Fig. 2b). A total of 25,512 unigenes were matched in 19 metabolic pathways and ‘translation’ (2562), ‘carbohydrate metabolism (2,215) and ‘folding, sorting and degradation’ (1,184) were the three richest pathways. In addition, 490 unigenes were mapped to the ‘Metabolism of terpenoids and polyketides’, including ‘Terpenoid backbone biosynthesis’ (ko00900, 147), ‘Monoterpenoid biosynthesis’ (ko00902, 12), ‘Sesquiterpenoid and triterpenoid biosynthesis’ (ko00909, 44), ‘Diterpenoid biosynthesis’ (ko00904, 58), ‘Limonene and pinene degradation’ (ko00903, 88), ‘Carotenoid biosynthesis’ (ko00906, 151), ‘Brassinosteroid biosynthesis’ (ko00905, 23) and ‘Zeatin biosynthesis’ (ko00908, 98).

Based on sequence homology, 51,768 annotated unigenes were categorized into three ontologies with 56 GO terms (Fig. 2c). Within the category of biological process (BP) category, genes matched to 25 GO terms, the most highly represented of which were ‘cellular process’ (28,998), ‘metabolic process’ (27,125) and ‘single-organism process’ (20,998). For the molecular function (MF) category, ‘binding’ (27,919) and ‘catalytic activity’ (22,472) were the two most abundant of 21 GO terms. The largest associated term within the 10 GO terms of the cellular component (CC) category was ‘cell’ (15,526).

### The identification of relative DEGs in *C. camphora* chemotypes and enrichment analysis of transcripts

**Fig. 3:**
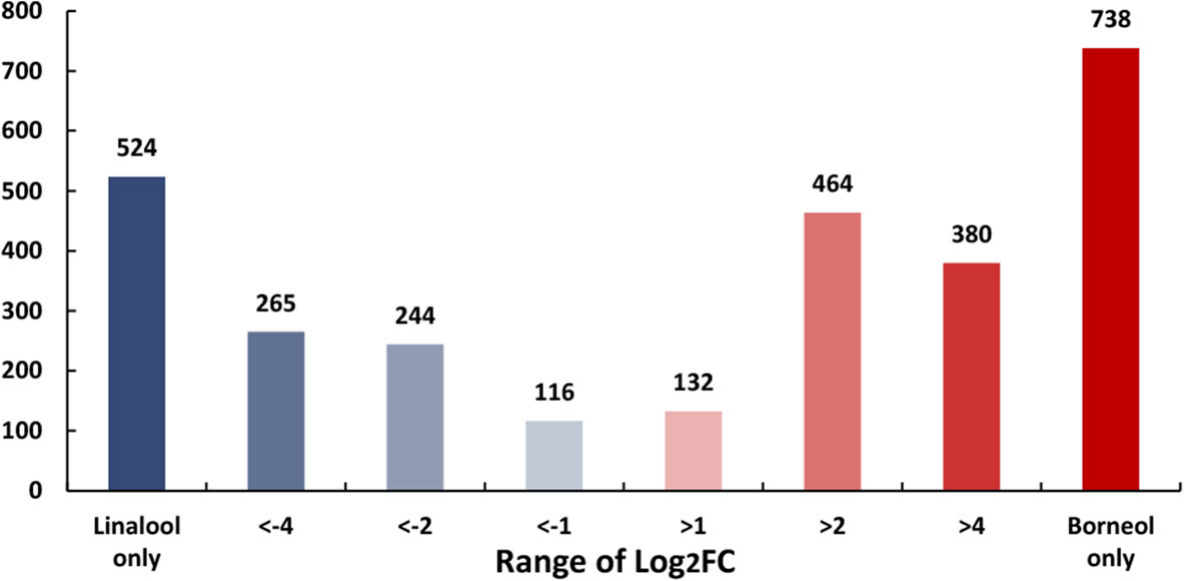
Number of and differentially expressed genes (DEGs) in Borneol-type and Linalool-type of *C. camphora*. Red: upregulated genes in the borneol-type; Blue: downregulated genes in the borneol-type. Relative expression of DEGs selected at Q-value < 0.05. The darker colors of bars represent larger change in expression. The fold change (FC) was calculated as the ratio between the borneol-type and linalool-type. The *x*- axis represents the range of Log_2_ FC. The *y*-axis indicates the number of detected DEGs

To fully explore potential differential gene expression between borneol and linalool chemotypes of *C. camphora*, the clean reads were mapped to the unigene database. The normalization of gene expression data was performed using multiple correction methods [42], and the differentially-expressed genes between the borneol-type and linalool-type were characterized by DESeq with *Q*-value < 0.05 and |log2.Fold_change| > 1, resulting in a total of 2863 unigenes identified, consisting of 1714 up-regulated and 1149 down-regulated genes in the borneol-type relative to the linalool-type, 738 unigenes of which were borneol-type unique, 524 unigenes of which were linalool-type unique, and 1601 mixed assembly unigenes in the two chemotypes. The distribution of these genes is shown in Fig. 3.

To explore the difference of metabolic pathways between borneol chemotype and linalool chemotype, enriched KEGG pathway analysis has been performed using 2863 differentially expressed genes. The top 20 over-presented KEGG pathways categories were presented as dot plots (Additional file 4). The most significantly enriched pathway for up-regulated DEGs in the borneol chemotype relative to the linalool type was the “monoterpenoid biosynthesis” pathway, with three DEGs annotated. For the up-regulated DEGs in linalool chemotype, the most significantly enriched pathway was “folate biosynthesis”.

**Fig. 4:**
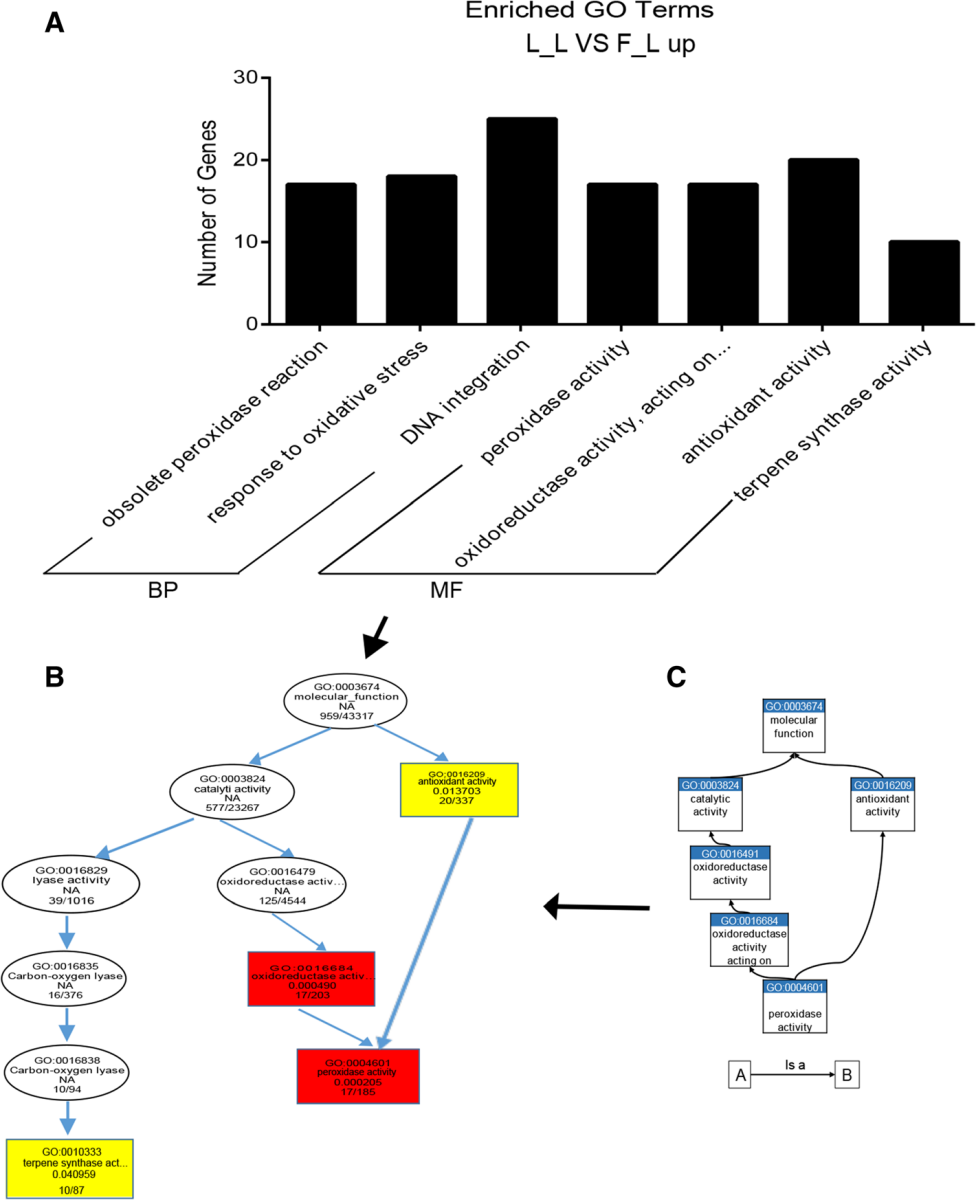
GO enrichment analysis of up-regulated DEGs. **a** GO enrichment histogram. The *x*- axis represent the enriched GO terms. The y-axis represents number of DEGs. **b** Thumbnails view of DAGs on BP and MF. The nodes are colored according to the *q*-value: red indicates high confidence level (*q*-value < 0.01), yellow indicates common confidence level (0.01 < *q*-value < 0.05), and blank nodes are associated terms. The node horizontal position indicates the depth of GO terms. In every node, four rows represent detailed information of GO ID, GO term, *q*-value, and DEG numbers with background gene numbers. **c** Original model of the GO ancestor chart. Terms linked with an arrow have a relationship of “Is a”, i.e., “term A is a term B” means that term A is a subtype of term B

In the GO enrichment analysis, 124 up-regulated DEGs between the borneol-type and linalool-type profiles were enriched and matched in 7 GO terms that belonged to biological process and molecular function (Fig. 4a). The structure of GO can be described in the form of a directed acyclic graph (DAG) in which each GO term is depicted as a node and the parentages as an arrow. Peroxidase activity and terpene synthase activity were significantly enriched (Fig. 4b).

### Candidate genes involved in terpenoid biosynthesis

**Fig. 5:**
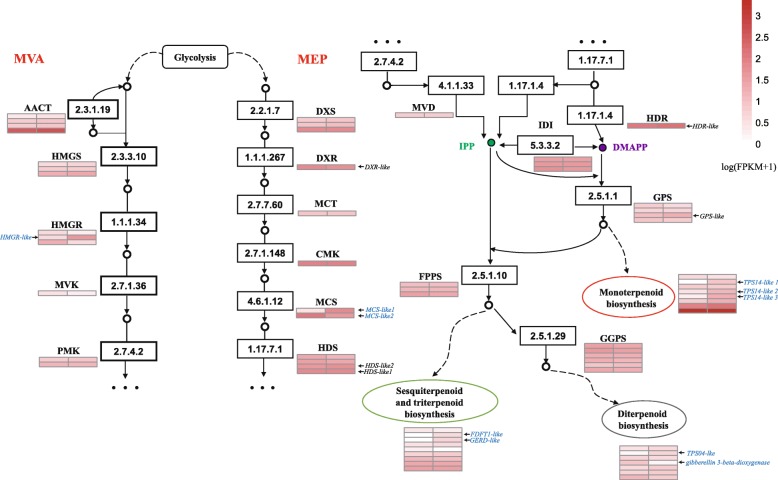
Differentially expressed genes in terpenoid backbone biosynthesis in *C. camphora*. Terpenoids biosynthesis pathway showing the subset of nodes or enzymes that constitute the process. Enzymes expression patterns are indicated at the side of each step with the value of log(FPKM+ 1). The expression pattern of each unigene is shown within two-column grids, with the left column representing the linalool-type and the right one representing the borneol-type. Significantly differentially expressed unigenes are blue. CCAT, Acetyl-CoA C-acetyltransferase; HMGS, hydroxymethylglutaryl-CoA synthase; HMGR, hydroxymethylglutaryl-CoA reductase (NADPH); MVK, mevalonate kinase; PMK, phosphomevalonate kinase; MVD, diphosphomevalonate decarboxylase; DXS, 1-deoxy-D-xylulose-5-phosphate synthase; DXR, 1-deoxy-D-xylulose-5-phosphate reductoisomerase; MCT, 2-C-methyl-D-erythritol 4-phosphate cytidylyltransferase; CMK, 4-diphosphocytidyl-2-C-methyl-D-erythritol kinase; MCS, 2-C-methyl-D-erythritol 2,4-cyclodiphosphate synthase; HDS, (E)-4-hydroxy-3-methylbut-2-enyl-diphosphate synthase; HDR, 4-hydroxy-3-methylbut-2-en-1-yl diphosphate reductase; IDI, isopentenyl-diphosphate Delta-isomerase; DMAPP, dimethylallyl diphosphate; GPS, geranyl diphosphate synthase; FPPS, farnesyl diphosphate synthase; GGPS, geranylgeranyl diphosphate synthase

To explore the regulatory mechanisms for the accumulation patterns of different terpenoids in *C.camphora*, the expression profiles of genes involved in terpenoid biosynthesis were analyzed. A total of 67 expressed unigenes encoding terpenoid biosynthesis enzymes were identified in *C. camphora* (Additional file 3). The expression data of all these unigenes is shown in Fig. 5, as well as the FPKM values in Additional file 5. Most of the genes encoding key enzymes in terpenoid backbone pathway (MEP and MVA pathway, KEGG entry ko00900) exhibited a high transcriptome expression level, were not differentially expressed between the two chemotypes except for one HMGR and two MCS unigenes. Interestingly, unigenes in the MEP pathway show a higher expression level than those of the MVA pathway (Fig. 5). Both MEP and MVA pathways generate IPP and its isomer DMAPP, which are precursors of the production of terpenoids. It is indicated that active biosynthesis of building blocks make contribution to a large accumulation of different class of terpenoids which is consistent with our composition analysis of leaf extract in *C. camphora* (Fig. 1c).

In addition, we characterized twenty-four unigenes encoding terpenoid synthase, including eight in monoterpenoid biosynthesis, seven in diterpenoid biosynthesis and nine in sesquiterpenoid and triterpenoid biosynthesis(Fig. 5). Among them, three monoterpenoid synthase unigenes, one diterpenoid synthase unigene and two unigenes involved in sesquiterpenoid and triterpenoid synthase were up-regulated in the borneol chemotype, while only one diterpenoid synthase unigenes were up-regulated in the linalool chemotype. Overall, our results of the high expression levels for the enzymes in terpenoid backbone pathway are consistent with a high rate of terpenoid synthesis, and suggest that the different expression level of TPS genes in the two chemotypes may be the reason for their different terpenoid compositions.

### qRT-PCR validation of DEGs from the RNA-Seq analysis

**Fig. 6:**
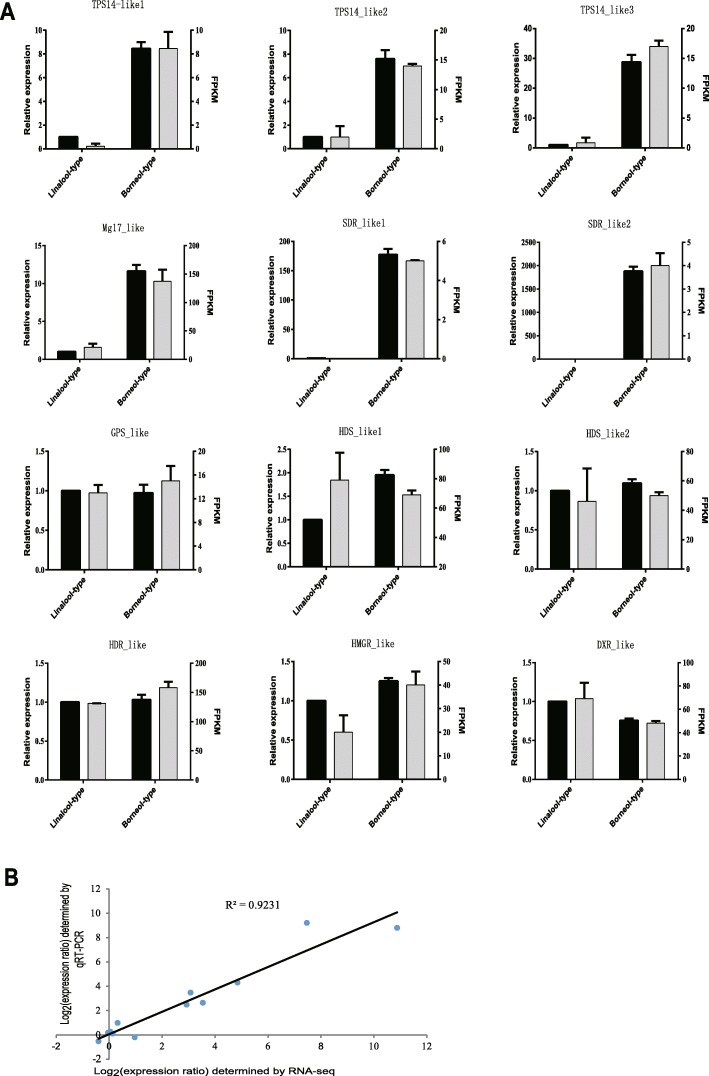
qRT-PCR validation of selected genes in linalool and borneol chemotypes of *C. camphora*. **a** The gray bars represent the relative expression determined with RT-qPCR (left y-axis) and the black bars represent the level of expression (FPKM) of the transcripts (right y-axis). The relative expression levels were estimated from the threshold of PCR cycle with the delta-delta CT method. The error bars indicate the standard errors from two biological and three technical replicates. **b** Scatter plots show simple linear regression and the R-squared (R^2^) between RNA sequencing data and qRT-PCR validation data expressed in terms of log_2_FC. The fold change (FC) was calculated as the ratio between the linalool-type and borneol-type of *C. camphora*

**Fig. 7:**
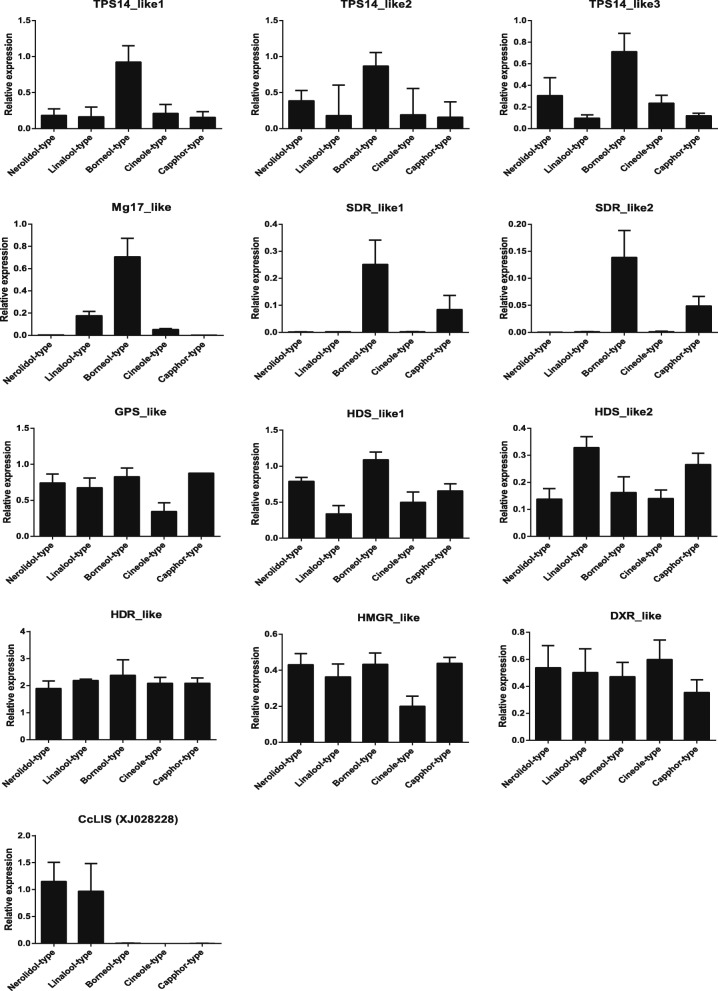
qRT-PCR validation of selected genes in five chemotypes of *C. camphora*. Five chemotypes include camphor-, cineole-, nerolidol-, borneol- and linalool-types. Relative expression levels were estimated from the threshold of the PCR cycle by the Delta CT method. The values indicate the means of two biological replications

To validate the expression patterns of terpenoid biosynthetic genes obtained from RNA-Seq analysis, qRT-PCR was conducted to examine the expression levels of twelve unigenes in the two chemotypes (Fig. 6). The expression levels of these selected genes from qRT-PCR analyses were generally consistent with those deduced from their fragments per kilobase per million mapped (FPKM) data from RNA-Seq (Fig. 6a). The correlation between the qRT-PCR and RNA-Seq measurements was evaluated and the coefficient of determination (or R-squared) was 0.9231(Fig. 6b). The obtained results confirm the reliability of the transcriptomic profiling data estimated from RNA-Seq data. Moreover, the expression of these genes has also been validated in three other chemotypes (camphor-, cineole- and nerolidol-types) by qRT-PCR except for borneol- and linalool-types of *C. camphora* (Fig. 7).

## Discussion

Monoterpenes are the main components in leaf extracts in many members of the genus *Cinnamomum*, such as *C. osmophloeum* [43], *C. burmannii* [44] and *C. kanehirae* Hay [45]. In this study, 24 components were identified in borneol and linalool-chemotypes of *C. camphora*. An analysis of the components showed that the essential oil or crystal isolated from *C. camphora* consists primarily of monoterpenes with very minor quantities of sesquiterpenes and diterpenes. However, the most prevalent monoterpene differed between the two chemotypes, with a high content of (−)-borneol in the borneol-type and a higher content of linalool in the linalool-type than in the borneol-type. Therefore, the different major monoterpenes mainly contributed to the differences between the essential oil of the two chemotypes.

In recent years, genes related to terpenoids biosynthesis have been extensively studied in different plants. Terpenoids are rich in the Lauraceae family, while only several genes involved in terpenoid biosynthesis have been successfully identified and functionally characterized so far [21, 43]. In *Litsea cubeba*, three TPS genes encoding monoterpenoid synthase enzymes were isolated and functionally catalyzed the formation of trans-ocimene, α-thujene and (+)-sabinene [46]. In *Laurus nobilis*, TPS enzymes which catalyze the formation of 1,8-cineole, cadinenes and geranyllinalool were characterized [47]. The bioinformatic analysis presented here aimed to discover genes involved in terpenes synthesis and their precursors in the MVA and MEP pathways. A total of 67 expressed unigenes likely involved in terpenoid biosynthesis were isolated from the transcriptome profile of *C. camphora*. Multiple unigenes were annotated as the same enzyme, and these unigenes may represent various alternatively spliced transcripts or members of a gene family.

The regulation of plant MVA and MEP pathway gene expression occurs mainly at the transcriptional level [48]. HMGR and DXS are two rate-limiting enzymes in the MVA [49] and MEP pathway, respectively [50]. Thirty expressed unigenes which were identified to be highly homologous with 14 known enzymes have been annotated into the MVA and MEP pathways of terpenoid backbone biosynthesis in *C. camphora*, and shown to exhibit a high transcriptome expression level in both two chemotypes. The MEP pathway provides precursors for the synthesis of monoterpenes and diterpenes in plastids, whereas sesquiterpenes are derived from precursors of the MVA pathway in the cytosol [48]. Cross talk between these two different terpene backbone pathways has been documented, whereas the relative contribution of each pathway to the biosynthesis of the various class of terpenes remain uncertain.

A large number of different TPS and the fact that some TPS produce multiple products are the chief reasons for the variety in terpenes [51]. Linalool synthase isolated from *C. osmophloeum* were able to generate the S-(+)-linalool from geranyl diphosphate (GPP) and (E)-nerolidol from farnesyl diphosphate (FPP) [43]. In addition, the products of TPS can be further modified by oxidation, peroxidation, methylation and acylation etc., such as by cytochrome P450 dependent monooxygenases and short-chain dehydrogenases/reductases (SDRs) [52]. In the GO enrichment analysis, 124 up-regulated DEGs between the borneol-type and linalool-type profiles were enriched, among these 10 and 17 DEGs were significantly enriched in the terpene synthase activity and oxidoreductase activity term of the DAG, respectively, indicating that terpene synthase activity and oxidoreductase activity were likely to be the terms leading to the differential accumulation of terpenoids in borneol and linalool chemotypes of *C. camphora*. In addition, 37 unigenes related to pathways of prenyl diphosphat, monoterpenoid, sesquiterpenoid and diterpenoid biosynthesis were identified in the current study. Mono-, sesqui−/tri-, and di-TPS have been synthesized by the activities of geranyl diphosphate synthase (GPS), farnesyl diphosphate synthase (FPPS) and geranylgeranyl diphosphate synthase (GGPPS) from GPP, FPP and geranylgeranyl diphosphate (GGPP), respectively [9]. Four GPS, three FPPS and six GGPS have been identified in both borneol and linalool chemotypes but with no difference in expression levels. In addition, a total of seven TPSs including three mono-TPSs, two di-TPSs and sesqui−/tri-TPSs were differentially expressed in the different chemotypes. This suggests that the different expression level of TPS genes in the two chemotypes may be the reason for their accumulation of different terpenoids.

In previous research, (+)-borneol as an intermediate product of camphor biosynthesis has been proved to be derived from the conversion of GPP to bornyl pyrophosphate (BPP) by the action of bornyl pyrophosphate synthetase (BPPS) and BPP was subsequently hydrolyzed to borneol [53]. BPPS, a metal-requiring monoterpene cyclase [54], has to date been cloned only from *Salvia officinalis* [53] and *Lavandula angustifolia* [55]. Linalool, an acyclic monoterpene, is a main product of linalool synthases and bi-functional synthases for both linalool and nerolidol production have been characterized in many plant species [51]. It is interesting that three of the mono-TPSs, *TPS14_like*, *TPS14_like2* and *TPS14_like3*, showed an up-regulated expression in the borneol-type compared to the linalool-type in this study, and their protein were aligned with some linalool synthases and borneol pyrophosphate synthases that have been published already (Additional file 6). However, TPS14 is a linalool synthase expressed in *Arabidopsis thaliana* flowers [56]*.* It has been reported that the specific biochemical functions of individual TPS family members cannot be predicted based on sequence similarity alone, as changes in only a few amino acids can lead to drastic changes in the terpenoid profile of a given TPS enzyme [18, 57]. In addition, many TPSs are multi-product enzymes and can often give rise to mixtures of the same compounds in differing proportions [58]. Furthermore, linalool biosynthesis can be altered by alternative transcript splicing of key biosynthetic enzymes in *Camellia sinensis* [59]. These make it difficult to determine transcript abundance of individual *TPS* unigenes using qRT-PCR. Nevertheless, *TPS14_like*, *TPS14_like2* and *TPS14_like3* may play an important role in terpenoid accumulation in the borneol-type of *C. camphora.* The specific function of these three genes remains to be further verified.

## Conclusion

In this study, we conducted leaf transcriptome and metabolic analysis between linalool- and borneol-chemotypes in *C. camphora*. This data provides a comprehensive coverage of terpenoid biosynthesis in *C. camphora*. Monoterpenes were identified as the major components in the leaf of the two chemotypes. Beta-linalool was the most abundant component in the linalool-chemotype while (−)-borneol was the major component in the borneol-chemotype. A comparison of the transcriptomes of these two chemotypes led to the identification of 2863 differentially expressed unigenes. Analysis of these unigenes provides an insight into the gene expression patterns and biological processes active in *C. camphora*. GO and KEGG enrichment analysis revealed that terpene synthase activity and oxidoreductase activity could explain the differential accumulation of terpenoids between borneol and linalool chemotypes in *C. camphora*. A total of 67 candidate unigenes were identified to be involved in terpenoid biosynthesis in *C. camphora*. Most notably, three unigenes involved in monoterpenoid biosynthesis were identified. Further functional studies are needed to elucidate regulatory mechanisms in the formation and accumulation of terpenoids. The transcriptome sequences and gene expression profile provide valuable information for understanding the accumulation of terpenoids in different chemotypes of *C. camphora*. The study also provides worthy resources for bioengineering and synthetic biology study of terpenoids in *C. camphora*.

## Additional files

Additional file 1: Composition of the leaf extracts of camphor-, cineole- and nerolidol-types *C. camphora*. (XLSX 11 kb)

Additional file 2: Primer sequences used for quantitative reverse transcription polymerase chain reaction (qRT-PCR). (XLSX 10 kb)

Additional file 3: Sequences of candidate unigenes were identified to be involved in terpenoid biosynthesis. (TXT 108 kb)

Additional file 4: Enriched KEGG pathway analysis of significantly differentially expressed genes between borneol- and linalool-chemotypes. F_L was linalool-type, L_L was borneol-type. The y- axis indicates the pathway name; the x-axis indicates the enrichment factor corresponding to the pathway. The Rich factor means that the ratio of the enriched DEGs number and the number of background genes in corresponding pathway. The greater the Rich factor, the greater the degree of enrichment. The *q*-value is represented by the color of the dot (scale provided to the right of each panel). The number of DEGs is represented by the size of the dots. (PDF 227 kb)

Additional file 5: FPKM values of terpenoid biosynthesis pathway unigenes. (XLSX 15 kb)

Additional file 6: Alignment and phylogenetic tree of the amino acid sequences of linalool and borneol pyrophosphate synthases. (A) Alignment of the amino acid sequences of linalool and borneol pyrophosphate synthases. LaLINs, (−)-(3S)linalool synthase, *Lavandula angustifolia*, ABB73045.1; MaLIS, linalool synthase, *Mentha aquatica*, AAL99381.1; SoSBS, (+)-bornyl diphosphate synthase, *Salvia officinalis*, AAC26017.1; AaQH, (3R)-linalool synthase, *Artemisia annua*, AAF13357.1; AaQH5, (3R)-linalool synthase *Artemisia annua*, AAF13356.1; LeMTS1, (−)-(3R)-Linalool synthase 1, *Solanum lycopersicum*, AAX69063.1; PaTPS-Lin, (−)-linalool synthase *Picea abies*, AAS47693.1; CcLIS, putative linalool synthase, *Cinnamomum camphora*, XJ028228; OsLIS, linalool synthase, *Oryza sativa*, ACF05530.1; CsNES/LIS, nerolidol/linalool synthase, *Camellia sinensis*, AGX26045.1; FaNES1, (+)-(3S)-Linalool synthase, *Fragaria* x *ananassa*, CAD57081.1; AmNES/LIS-2, nerolidol/linalool synthase, *Antirrhinum majus*, ABR24418.1; AtLIS, linalool synthase, *Arabidopsis thaliana*, AAO85533.1; ObLIS, R-linalool synthase, *Ocimum basilicum*, AAV63789.1; CbLIS, linalool synthase, *Clarkia breweri*, AAD19840.1. (B) Phylogenetic analysis by Maximum likelihood method. Evolutionary analyse were coducted in MEGA7. (PDF 209 kb)

## Abbreviations

bp: Base pairs (measuring unit)
DAG: Directed acyclic graphs
DEGs: Differentially expressed genes
DMAPP: Dimethylallyl diphosphate
FPKM: Fragments per kilobase of exon per million fragments mapped
FPP: Farnesyl diphosphate
FPPS: Farnesyl diphosphate synthase
FW: Fresh weight
GC-MS: Gas chromatography-mass spectrometry
GGPP: Geranylgeranyl diphosphate
GGPS: Geranylgeranyl diphosphate synthase
GO: Gene ontology
GPP: Geranyl diphosphate
GPS: Geranyl diphosphate synthase
IPP: Isopentenyl diphosphate
KEGG: Kyoto encyclopedia of genes and genomes
KO: KEGG ortholog database
KOG: EuKaryotic Orthologous Groups
MEP: 2-C-methyl-D-erythritol 4-phosphate
MVA: Mevalonate acid
Nr: NCBI non-redundant protein sequences
Nt: NCBI non-redundant nucleotide sequences
Pfam: Protein family
qRT-PCR: Quantitative reverse transcription PCR
RNA-Seq: RNA-sequencing
Swiss-Prot: A manually annotated and reviewed protein sequence database
TPS: Terpene synthases
