# Supplementary figures and images for: Transcriptome analysis and identification of genes related to terpenoid biosynthesis in Cinnamomum camphora
Source: BMC Genomics. 2018 Jul 24;19:550. doi: 10.1186/s12864-018-4941-1 (PMC6057064; doi:10.1186/s12864-018-4941-1)

A

DOWN-DEG-F\_LvsL\_L

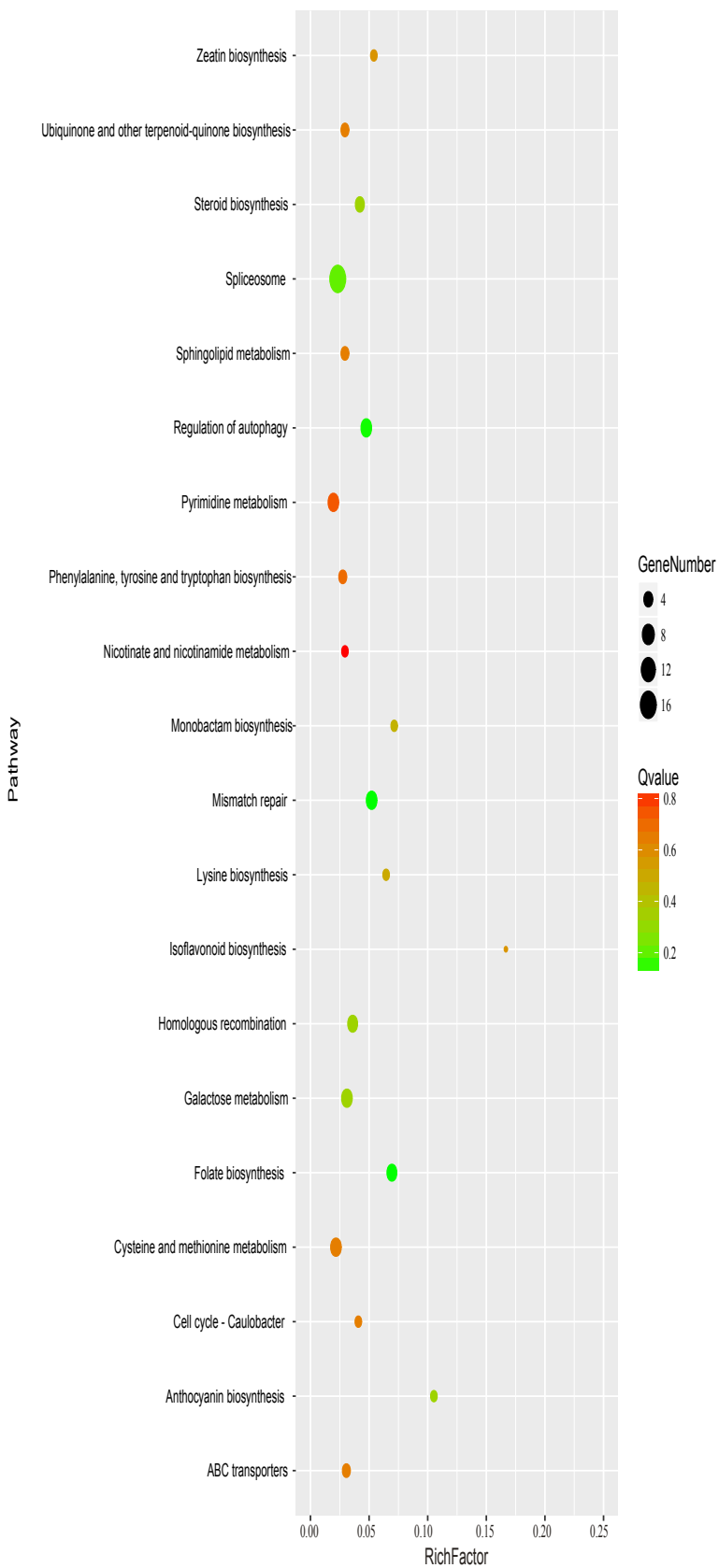

B

UP-DEG-F\_LvsL\_L

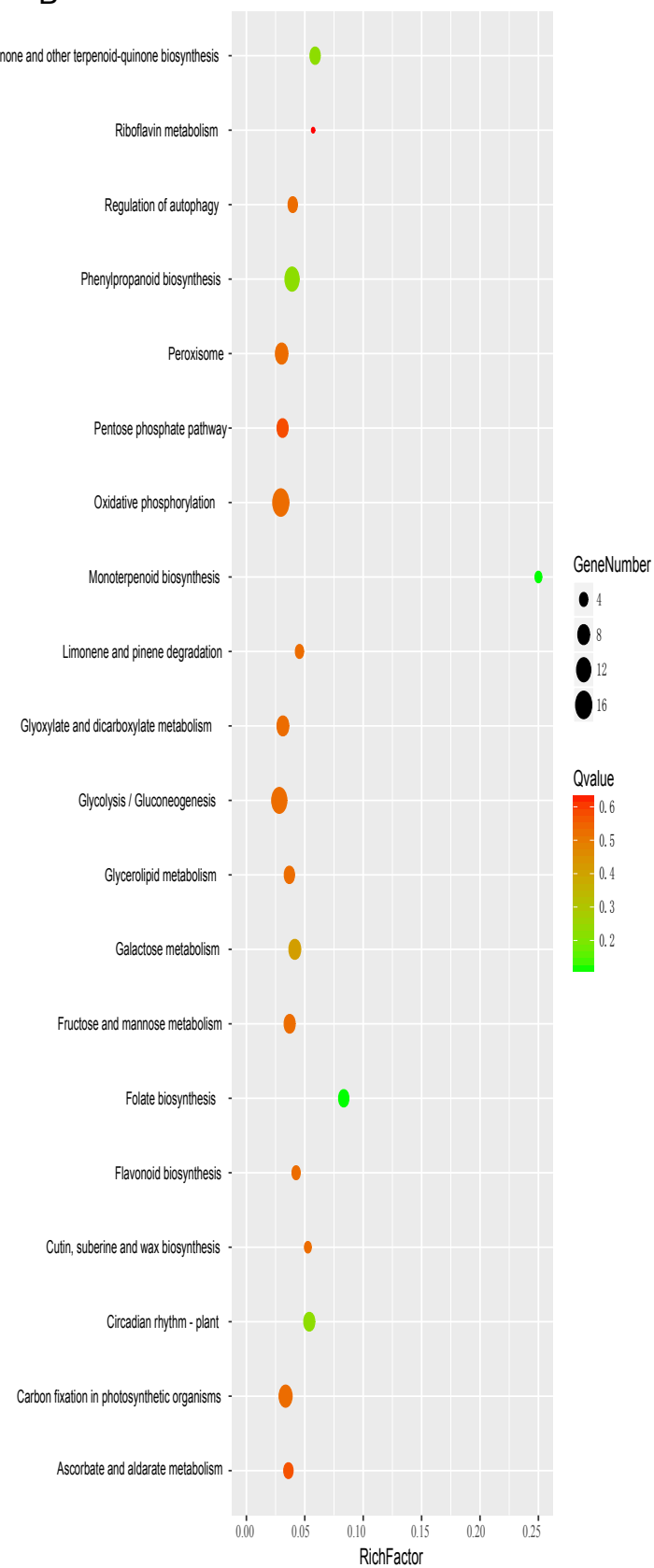

Supplement: Supplementary file 4 — Enriched KEGG pathway analysis of significantly differentially expressed genes between borneol- and linalool-chemotypes. F_L was linalool-type, L_L was borneol-type. The y- axis indicates the pathway name; the x-axis indicates the enrichment factor corresponding to the pathway. The Rich factor means that the ratio of the enriched DEGs number and the number of background genes in corresponding pathway. The greater the Rich factor, the greater the degree of enrichment. The q-value is represented by the color of the dot (scale provided to the right of each panel). The number of DEGs is represented by the size of the dots. (PDF 227 kb) [file 12864_2018_4941_MOESM4_ESM.pdf]

A

CLUSTAL 2.1 MULTIPLE SEQUENCE ALIGNMENT

File: D:/LIS and BPPS\_protien.ps  
Page 1 of 2

Date: Tue May 29 11:36:26 2018

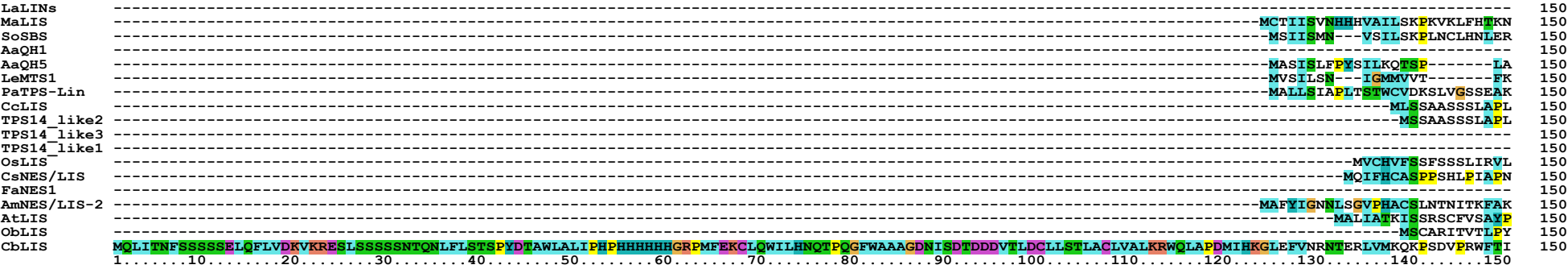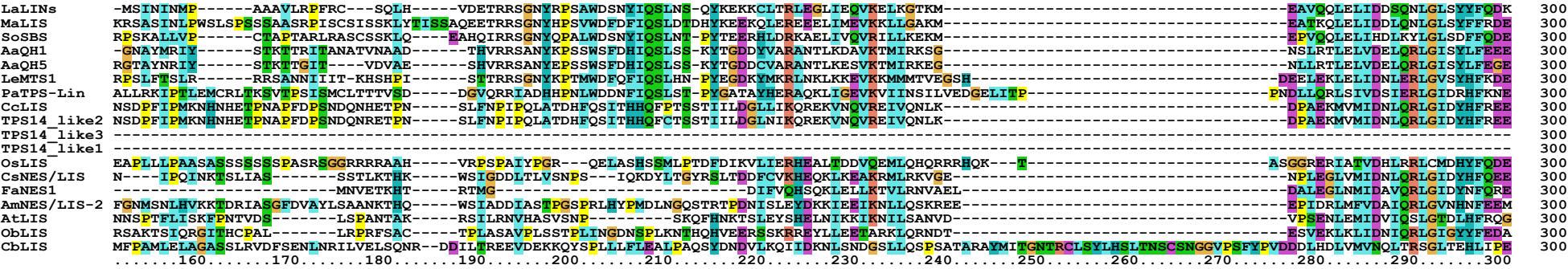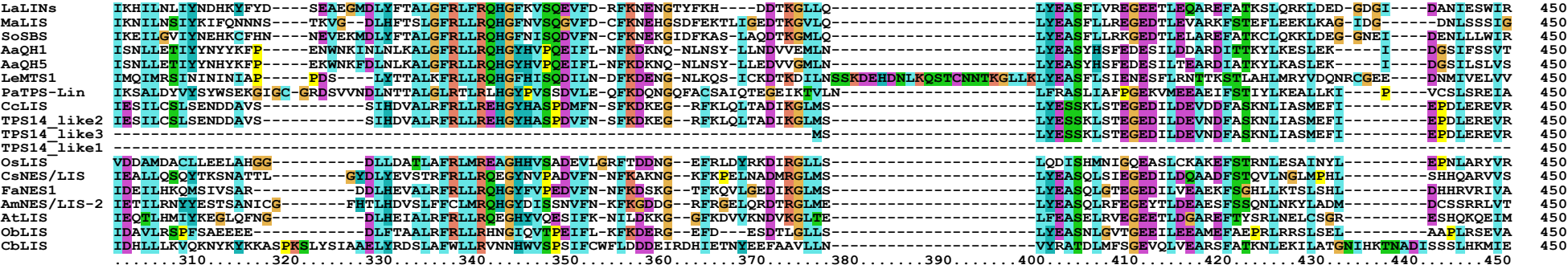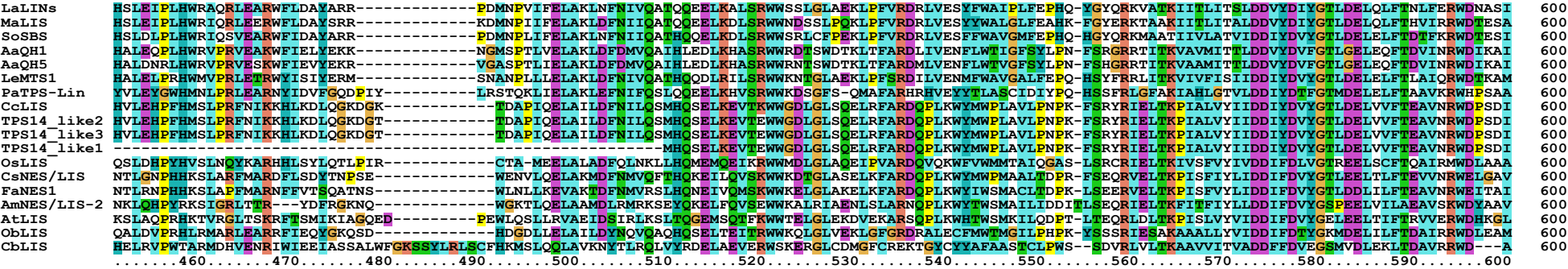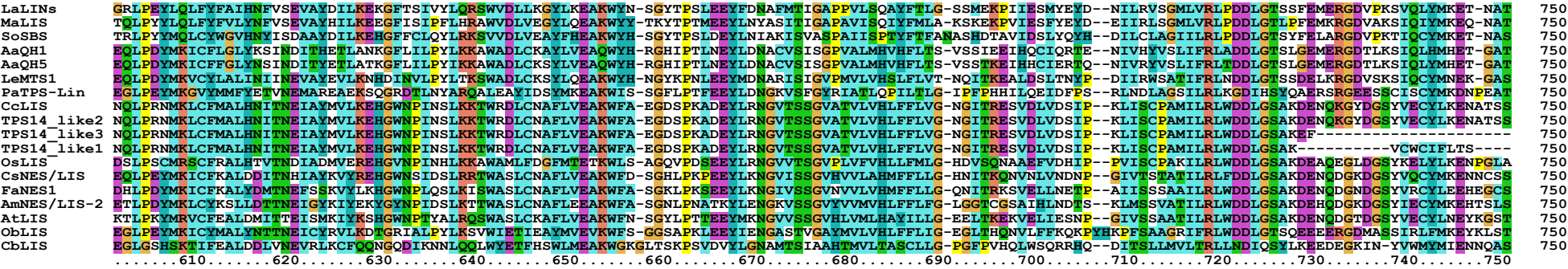

B

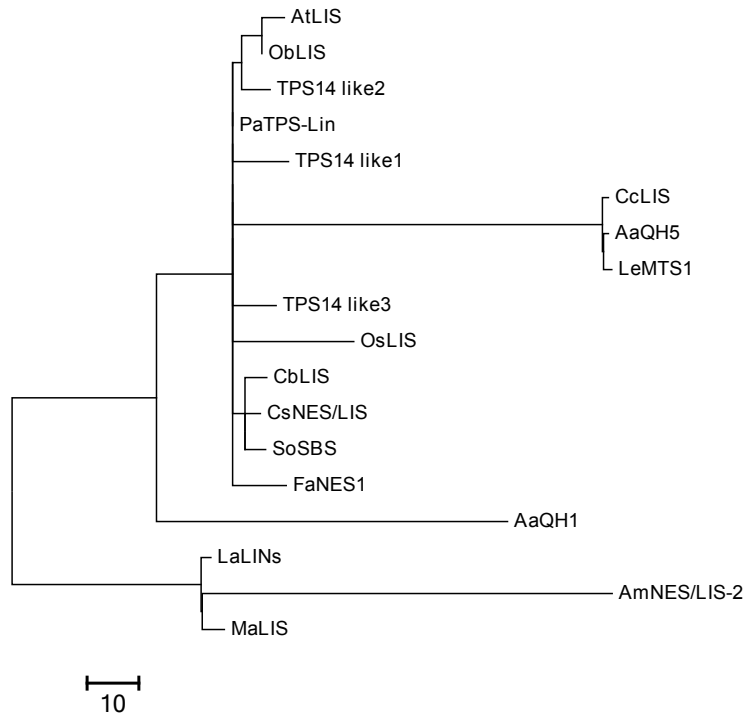

Supplement: Supplementary file 6 — Alignment and phylogenetic tree of the amino acid sequences of linalool and borneol pyrophosphate synthases. (A) Alignment of the amino acid sequences of linalool and borneol pyrophosphate synthases. LaLINs, (−)-(3S)linalool synthase, Lavandula angustifolia, ABB73045.1; MaLIS, linalool synthase, Mentha aquatica, AAL99381.1; SoSBS, (+)-bornyl diphosphate synthase, Salvia officinalis, AAC26017.1; AaQH, (3R)-linalool synthase, Artemisia annua, AAF13357.1; AaQH5, (3R)-linalool synthase Artemisia annua, AAF13356.1; LeMTS1, (−)-(3R)-Linalool synthase 1, Solanum lycopersicum, AAX69063.1; PaTPS-Lin, (−)-linalool synthase Picea abies, AAS47693.1; CcLIS, putative linalool synthase, Cinnamomum camphora, XJ028228; OsLIS, linalool synthase, Oryza sativa, ACF05530.1; CsNES/LIS, nerolidol/linalool synthase, Camellia sinensis, AGX26045.1; FaNES1, (+)-(3S)-Linalool synthase, Fragaria x ananassa, CAD57081.1; AmNES/LIS-2, nerolidol/linalool synthase, Antirrhinum majus, ABR24418.1; AtLIS, linalool synthase, Arabidopsis thaliana, AAO85533.1; ObLIS, R-linalool synthase, Ocimum basilicum, AAV63789.1; CbLIS, linalool synthase, Clarkia breweri, AAD19840.1. (B) Phylogenetic analysis by Maximum likelihood method. Evolutionary analyse were coducted in MEGA7. (PDF 209 kb) [file 12864_2018_4941_MOESM6_ESM.pdf]
